# miR-103 Promotes Proliferation and Metastasis by Targeting KLF4 in Gastric Cancer

**DOI:** 10.3390/ijms18050910

**Published:** 2017-04-26

**Authors:** Jie Zheng, Yuzhen Liu, Yanchun Qiao, Liying Zhang, Shijun Lu

**Affiliations:** 1 Department of Diagnostic Pathology, Weifang Medical University, Weifang 261053, China; yuzhenliu2012@sina.com (Y.L.); haiyanzhang2012@sina.cn (L.Z.); 2 Department of Pathology, Affiliated Hospital of Weifang Medical University, Weifang 261053, China; qiaoyanchun2006@sina.com

**Keywords:** gastric cancer, microRNA-103, *KLF4*, proliferation, metastasis

## Abstract

MicroRNAs (miRNAs) play important roles in the cancer development and progression; overexpression of miR-103 has been identified in various tumors. However, its biological function and regulatory mechanism involved in modulation of human gastric cancer (GC) remain largely unknown. This study aimed to confirm clinical significance of miR-103 and investigate its biological role and underlying mechanism in GC. Real-time quantitative PCR (qRT-PCR) revealed miR-103 was highly expressed in GC tissues and cell lines. miR-103 expression was correlated closely with tumor size, Lauren’s classification, and lymph node metastasis. Importantly, Kaplan-Meier analysis revealed that high expression of miR-103 was significantly associated with poor overall survival and disease-free survival of GC patients. Downregulation of miR-103 by transfecting with miR-103 inhibitor significantly suppressed cell proliferation, induced apoptosis, inhibited migration and invasion in vitro and in vivo. Furthermore, miRNA target databases and luciferase reporter assay confirmed that Krüppel-like Factor-4 (*KLF4*) was a direct target of miR-103 in GC, and there was a significant inverse correlation between miR-103 and *KLF4* expression in GC tissues. Moreover, *KLF4* downregulation could rescue miR-103’s oncogenic effect on GC cell proliferation, apoptosis, migration, and invasion. Therefore, these results suggested that miR-103 overexpression could contribute to tumor progression by suppressing *KLF4*, and it might serve as a promising candidate for the prognosis of GC patients.

## 1. Introduction

Gastric cancer (GC) is the fourth most common malignant carcinomas and the second most frequent cause of cancer-related death worldwide [1,2]. Despite the considerable improvement in surgical and chemotherapeutic treatment, the prognosis of GC patients in advanced stage with lymph node metastasis still remains poor. The molecular mechanisms underlying gastric cancer development still need to be explored, allowing for the identification of novel therapeutic targets or prognostic markers.

MicroRNAs (miRNAs) are small, endogenous, non-coding RNAs about 20–25 nucleotides in length that can regulate target gene expression at the post-transcriptional level by inhibiting translation or/and cleaving the targeted mRNA [3] by binding to the 3′-untranslated regions (3′-UTRs) of target mRNA. In GC, investigations on miRNAs and their functions have provided new targets for therapeutic strategies.

Recently, miR-103 has been the focus of many studies and has been shown to function as a oncogen, and be upregulated in several types of cancer such as colorectal cancer [4], endometrial cancer [5], and prostate cancer [6]. The aberrant overexpression of miR-103 has been identified in gastric cancer by miRNA microarray and qRT-PCR [7,8,9,10]. miR-103 was identified as one of the most important microRNAs associated with GC progression [7]. Kim et al found that the expression of miR-103 was upregulated in gastric cancer and associated with the more advanced tumor stages, especially the invasion and metastasis using qRT-PCR [9]. Later studies suggested that miR-103 was significantly overexpressed in sera of both early and advanced-stage DGC (Diffuse-type gastric cancer)-bearing mice compared with corresponding controls by miRNA microarray and qPCR analyses [10]. However, the roles of miR-103 in the progression of GC and its underlying function to regulate tumor proliferation and metastasis are poorly understood, the clinical significance of miR-103 in the prognosis of patients with GC remains unclear.

This study investigated the expression levels of miR-103 in GC tissues and cells and assessed the correlation between clinicopathologic characteristics and miR-103 expression. The effect of miR-103 on biological behaviors, including cells apoptosis, proliferation, migration and invasion of GC, was also detected. Moreover, the mechanisms underlying the role of miR-103 in gastric cancer progression were also investigated.

## 2. Results

### 2.1. Upregulation of miR-103 in Gastric Cancer Tissues and Cell Lines

**Figure 1:**
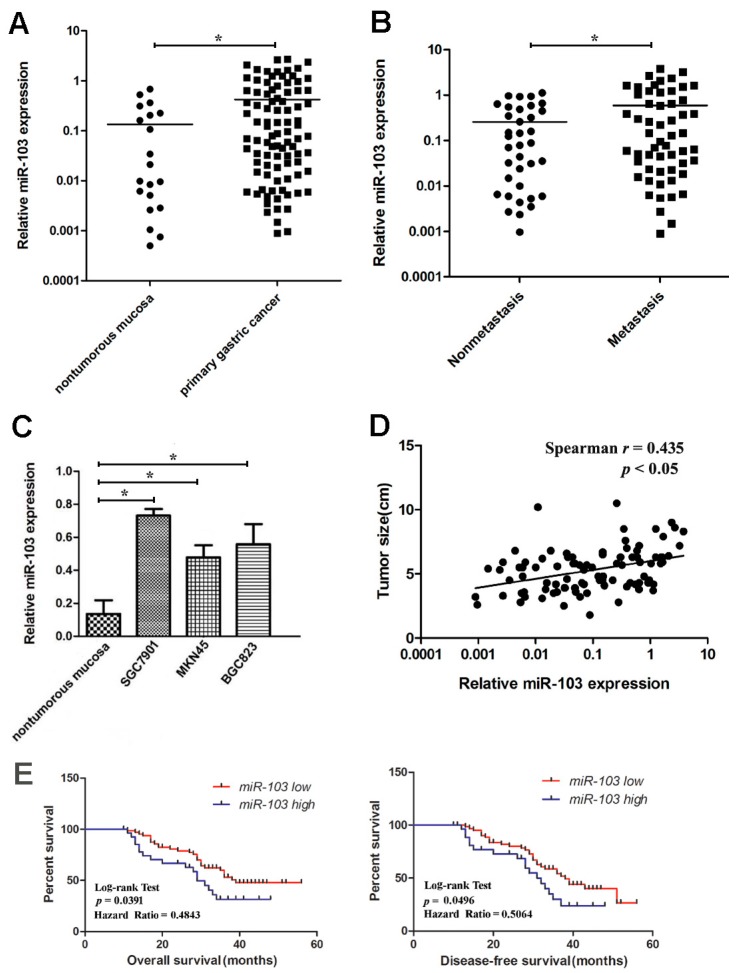
miR-103 is overexpressed in gastric cancer primary tumor tissues and cell lines and correlates with patient survival. (**A**) The miR-103 expression in 92 cases of primary GC tissues compared with 20 cases of adjacent nontumorous tissues analyzed by RT-qPCR; (**B**) The miR-103 expression in primary GC tissues with lymph node metastasis compared with those without lymph node metastasis; (**C**) miR-103 expression in gastric cancer cell lines; (**D**) miR-103 expression was positively associated with tumor size by Spearman’s correlation test (*n* = 92) and (**E**) The correlation between miR-103 expression and GC patient survival. * *p* < 0.05; the data represent the mean ± standard deviation (SD) from triplicate measurements.

This study investigated miR-103 expression by qRT-PCR in gastric cancer and adjacent nontumorous tissues. The miR-103 expression in tumor samples was significantly higher than that in adjacent nontumorous tissues (Figure 1A). The expression of miR-103 in patients with lymph node metastasis was significantly higher than those without lymph node metastasis (Figure 1B). This result was confirmed in GC cell lines, miR-103 was over-expressed in gastric cancer cell line than nontumorous mucosa, especially in the metastatic SGC7901 gastric cancer cell line (Figure 1C). To further investigate the clinicopathologic significance of miR-103 levels in GC samples, 92 GC patients were divided into two groups based on miR-103 expression level including the low miR-103 expression group (below the median value) and the high miR-103 expression group (above the median value), for survival analysis. Clinicopathologic analysis showed that the overexpression of miR-103 was correlated with the tumor size, Lauren’s classification and lymph node metastasis (Table 1). Moreover, spearman correlation test showed that miR-103 was positively related with tumor size (Figure 1D).

### 2.2. Overexpression of miR-103 Is Correlated with Poor Prognosis for Gastric Cancer

Kaplan–Meier analysis and log-rank test were used to evaluate the prognostic significance of miR-103 expression in GC. High miR-103 expression showed significantly shorter overall survival (OS) and disease free survival (DFS) of patients than that of patients with low miR-103 expression level (Figure 1E), indicating that miR-103 might serve as a promising candidate for the prognosis of GC patients.

### 2.3. Downregulation of miR-103 Impaired Proliferation and Induced Apoptosis of SGC7901 and BGC823 Cells

**Figure 2:**
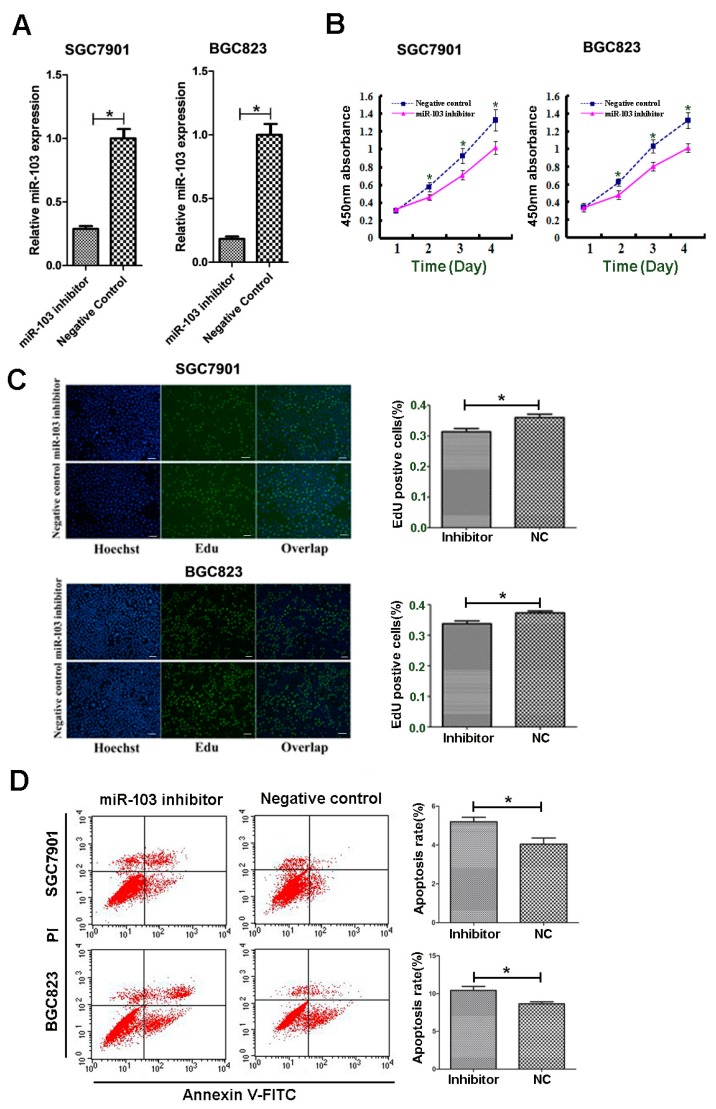
miR-103 promotes cell proliferation and reduced apoptosis of GC cells. (**A**) Analyses of miR-103 expression after transfection in SGC7901 and BGC823 cells by real-time PCR; (**B**,**C**) Influence of miR-103 downregulation on cell proliferation of SGC7901 and BGC823 cells by CCK-8 (**B**) and EdU assays (**C**,**D**) Influence of miR-103 knockdown on cell apoptosis. * *p* < 0.05, Scale bar = 100 μm for (**C**); the data represent the mean ± SD from triplicate measurements.

As shown in Figure 1C, SGC7901 and BGC823 showed relatively higher miR-103 expression. miR-103 inhibitor transfection was performed in these two cell lines. To monitor transfection efficiency, qRT-PCR was performed to determine miR-103 expression at 48 h after transfection. Relative miR-103 expression was significantly lower in SGC7901 and BGC823 cells transfected with miR-103 inhibitor than that in negative control (NC) groups (Figure 2A).

To explore whether miR-103 could influence GC cells proliferation, CCK-8 and EdU assays were used to assess cell growth ability. The results showed that downregulation of miR-103 could result in decreased growth rate of SGC7901 and BGC823 cells (Figure 2B,C). Additionally, it was found that miR-103 inhibitor, compared with the negative control, induced the apoptosis rate of SGC7901 and BGC823 cells (Figure 2D).

### 2.4. Reduction of miR-103 Inhibited GC Cells Migration and Invasion

**Figure 3:**
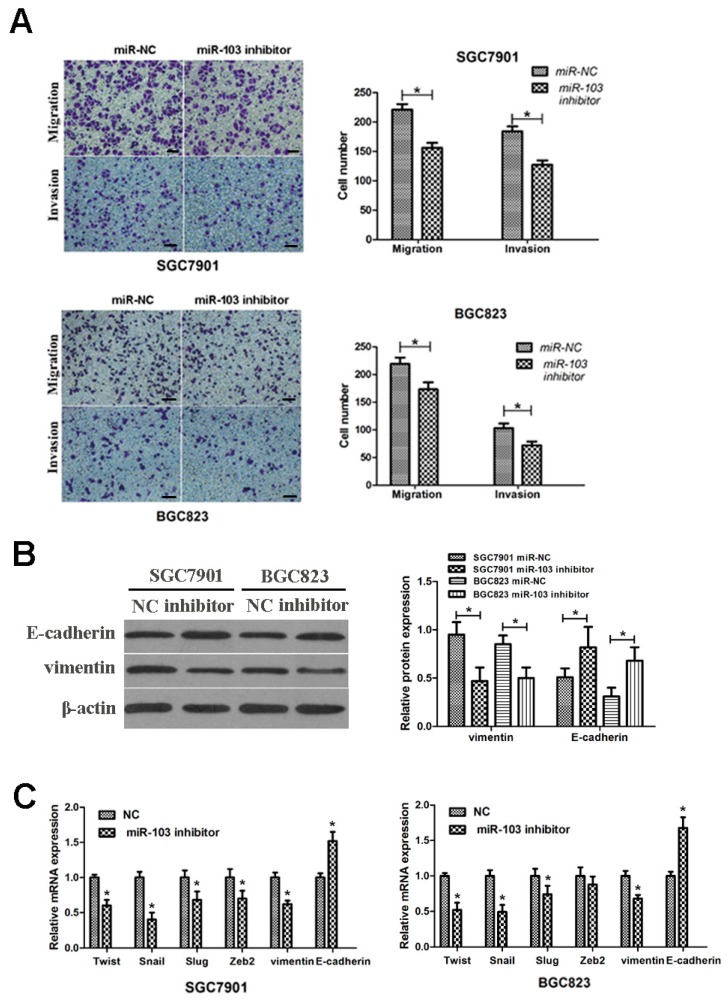
miR-103 promotes cell migration, invasion and mesenchymal-epithelial transformation (EMT) of GC cells. (**A**) Effect of miR-103 knockdown on cell migration and invasion ability in SGC7901 and BGC823 cells, Scale bar = 50 μm; (**B**) E-cadherin and vimentin expression in SGC7901 and BGC823 cells by western blot and (**C**) epithelial-to-mesenchymal transition (EMT)–associated genes expression in SGC7901 and BGC823 cells by RT-qPCR. * *p* < 0.05; the data represent the mean ± SD from triplicate measurements.

The effects of miR-103 on cell migration and invasion were assessed with transwell assays. When miR-103 expression was knocked down, reduced cell migration and invasion capability were shown in SGC7901 and BGC823 cells (Figure 3A).

### 2.5. Knockdown of miR-103 Suppressed the Epithelial–Mesenchymal Transition (EMT) Process of GC Cells

To investigate whether miR-103 is involved in EMT process of GC cells, the expression of a variety of EMT markers was detected. Downregulation of miR-103 increased the E-cadherin expression level and decreased the level of vimentin in GC cells (Figure 3B). Most of the EMT-associated genes tested were downregulated by miR-103 knockdown, with *Snail* being the most severely affected in SGC7901 and BGC823 cells (Figure 3C). Taken together, these findings demonstrated that knockdown of miR-103 could inhibit EMT in GC cells.

### 2.6. Downregulation of miR-103 Inhibited GC Growth and Lung Metastasis In Vivo

**Figure 4:**
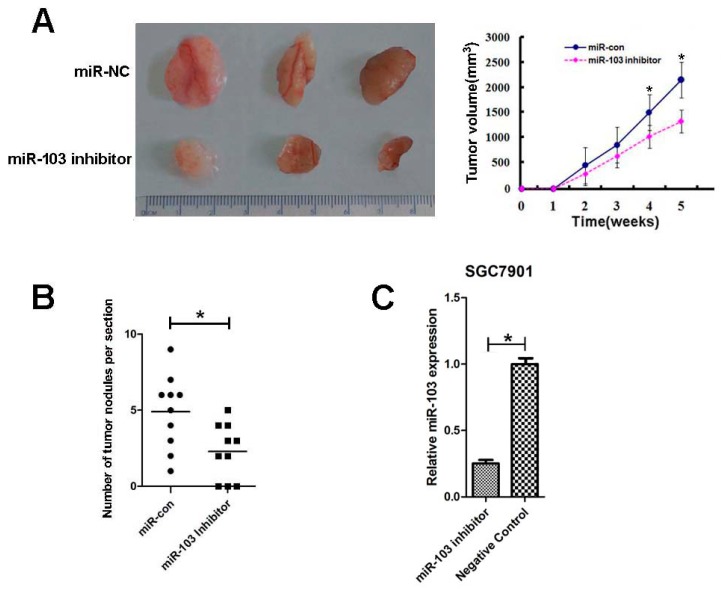
miR-103 promotes gastric cancer cell growth and metastasis in vivo. (**A**) Tumor volumes were measured on the indicated days; (**B**) The number of lung metastasis of indicated SCID mice groups and (**C**) RT-qPCR analysis of miR-103 expression in implanted tumors. * *p* < 0.05; the data represent the mean ± SD from triplicate measurements.

The growth of SGC7901 xenograft was significantly inhibited by knockdown of miR-103 (Figure 4A). The lung metastases of SGC7901 xenograft were also suppressed by downregulation of miR-103, and the number of lung metastatic nodules was decreased compared to the negative controls (Figure 4B). Meanwhile, RT-qPCR analysis showed that relative miR-103 expression of the tumor xenograft was lower in SGC7901 cells transfected with lentivirus miArrest™ miR-103 inhibitor than that in negative control groups (Figure 4C).

### 2.7. miR-103 Directly Targets and Down-Regulates KLF4 in GC Cells

**Figure 5:**
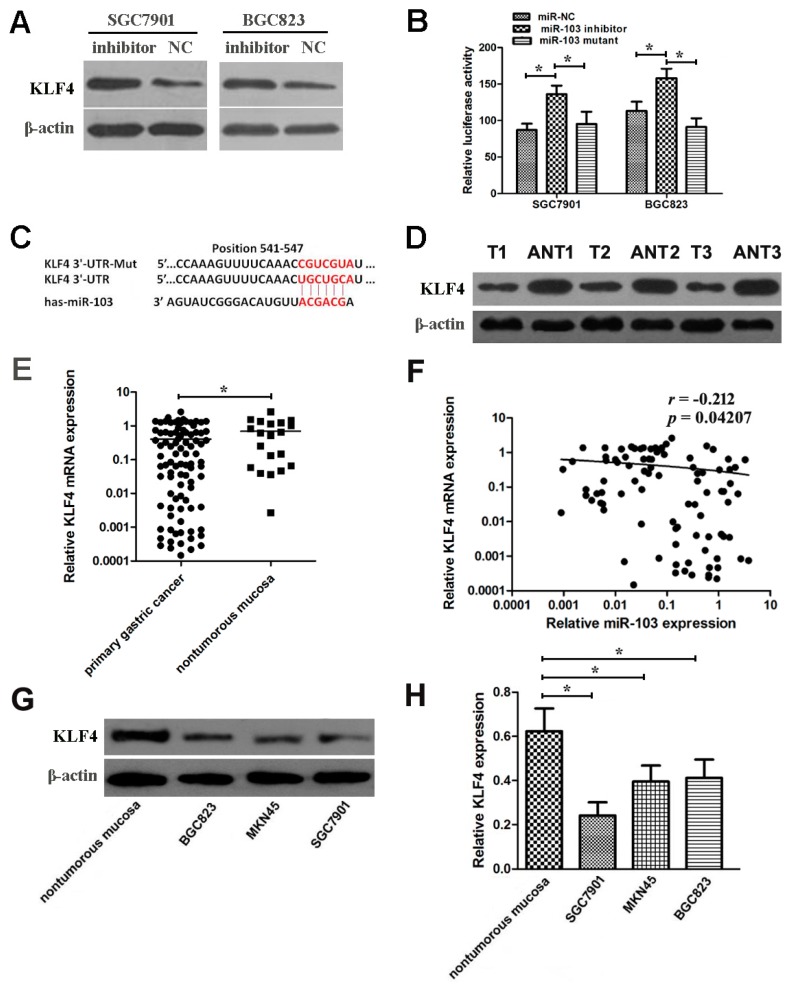
*KLF4* is a direct target of miR-103 and *KLF4* expression is inversely correlated with miR-103 expression in GC tissues. (**A**) Western blot analysis of KLF4 protein expression after transfection in SGC7901 and BGC823 cells; (**B**) Luciferase activities of wild-type and the mutant pmirGLO-*KLF4*-3′-UTR reporter in SGC7901 and BGC823 cells; (**C**) The predicted miR-103 binding site on the *KLF4* mRNA 3′-UTR and the corresponding mutations in 3′-UTR of *KLF4*; (**D**,**E**) KLF4 expression on protein level (**D**) and mRNA level (**E**) was determined in GC tissues (T) and adjacent nontumorous tissues (ANT); (**F**) Spearman’s correlation analysis was performed to detect the association between the expression level of miR-103 and *KLF4* in GC tissues; (**G**,**H**) KLF4 expression on protein level (**G**) and mRNA level (**H**) was determined in three GC cell lines (BGC823, SGC7901 and MKN45) and nontumorous mucosa. Error bars represent mean ± SD from three independent experiments. * *p* < 0.05.

To investigate the molecular mechanism underlying the tumor promoting function of miR-103, four cancer-associated genes (*DICER1*, *CPEB3*, *FOXP1*, and *KLF4*) predicted by three common databases (Pictar, Miranda, and Targetscan) were selected as candidates to be investigated. As shown in Figure 5A, Western blot analysis showed that the expression of KLF4 protein was significantly increased in SGC7901 and BGC823 cells when miR-103 inhibitor was transfected compared with the negative control. Previous study also reported that *KLF4* was a proliferation- and metastasis-associated gene in gastric cancer. Based on these results, we hypothesized that *KLF4* was a target of miR-103 in gastric cancer.

Consistent with the Western blot results, it was found that when miR-103 inhibitor was transfected, the activity of a luciferase reporter gene fused to the *KLF4* 3′-UTR was increased by 36.02 ± 1.4% and 28.48 ± 1.8% in SGC7901 and BGC823 cells compared with negative control groups (Figure 5B). To confirm that *KLF4* is a direct target of miR-103, *KLF4* 3′-UTR and the mutant counterparts were introduced into pmirGLO luciferase reporter plasmid. Downregulation of miR-103 increased luciferase activity in SGC7901 cells transfected with the wild-type 3′-UTR of *KLF4* but not in SGC7901 cells with mutant 3′-UTR (Figure 5B). Similar results were observed in BGC823 cells (Figure 5B). These data suggested that *KLF4* was a direct target of miR-103 in gastric cancer, and the position of 541-547 in the 3′-UTR of *KLF4* is the miR-103 binding site (Figure 5C).

### 2.8. miR-103 and KLF4 Are Clinically Relevant in Human GC Cell and Tissues

To elucidate the clinical relevance of miR-103 and *KLF4* in GC, the *KLF4* expression on mRNA level and protein level in T and ANT tissues were detected by western blot and qRT-PCR analysis, respectively. These results showed that *KLF4* expression was downregulated in GC (Figure 5D,E). Furthermore, there was an inverse association between the miR-103 and *KLF4* expression levels (*r* = −0.212, Figure 5F). In addition, the expression of *KLF4* in GC cell lines was assessed. Western blot and qRT-PCR analysis demonstrated that *KLF4* expressed at lower levels in GC cell lines compared with nontumorous mucosa (Figure 5G,H). These results further confirmed that *KLF4* expression was negatively regulated by miR-103 in GC. These data suggested that the inhibitory effect of miR-103 on the *KLF4* is clinically relevant in GC.

### 2.9. Overexpression of KLF4 Could Rescue the Oncogenic Effects of miR-103 in GC

**Figure 6:**
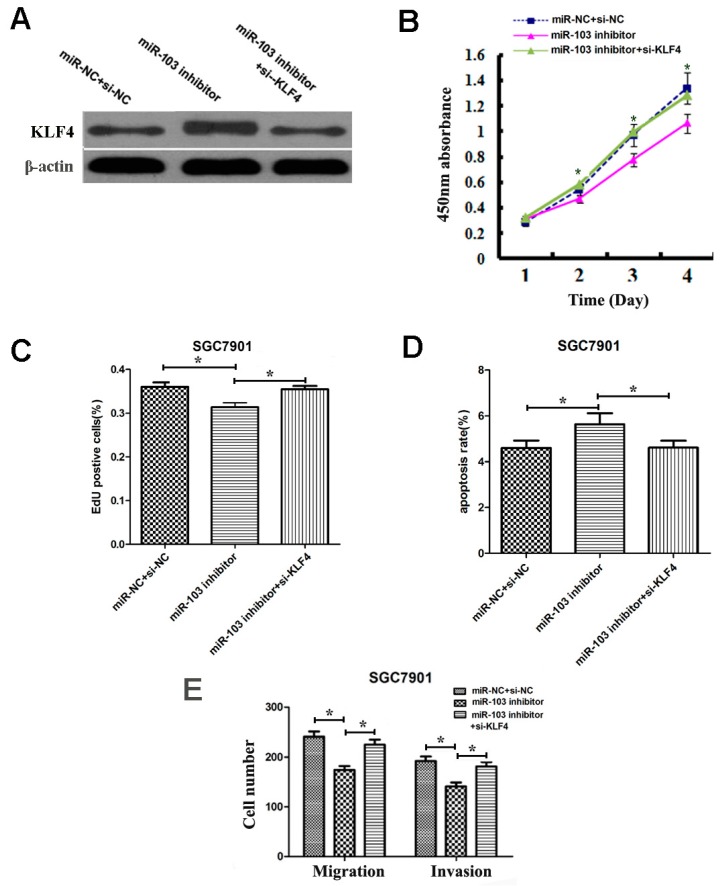
Downregulation of *KLF4* rescues miR-103’s oncogenic effect on GC cell proliferation, apoptosis, migration, and invasion in SGC7901cells. (**A**) KLF4 protein expression was detected in SGC7901 cells co-transfected with miR-103 inhibitor/miR-NC and *KLF4* siRNA or si-NC; (**B**–**E**) Cell proliferation, apoptosis, migration and invasion were assessed in SGC7901 cells co-transfected with miR-103 inhibitor/miR-NC and *KLF4* siRNA or si-NC. * *p* < 0.05; the data represent the mean ± SD from triplicate measurements.

To evaluate if *KLF4* is responsible for the functional effects of miR-103 in GC cells, rescue experiments were performed. SGC-7901 cells were transfected with the miR-103 inhibitor or miR-NC and *KLF4* siRNA. The transfection efficiency was verified by western blot (Figure 6A). Then cell proliferation, apoptosis, migration, and invasion assays were performed in above cells. The results showed that *KLF4* downregulation reversed the miR-103-mediated oncogenic influence on cell proliferation, migration, apoptosis, migration and invasion in SGC-7901 cells (Figure 6B–E). These results clearly demonstrated that miR-103 promotes cell proliferation, migration, and invasion, and inhibits apoptosis in GC cells, at least in part by targeting *KLF4*.

## 3. Discussion

miRNAs can be tumor suppressive or oncogenic in human malignant tumors by targeting downstream genes and thus is involved in the progression and metastasis of human tumors. In our study, we showed that miR-103 was overexpressed in primary GC tissues and cell lines. Further analysis demonstrated that miR-103 levels were even higher in these patients with LNM and larger tumor size (≥5cm). Spearman’s correlation test also confirmed that miR-103 expression was positively associated with tumor size. These results demonstrated that miR-103 might play an important role in regulating proliferation and metastasis of gastric cancer. Downregulation of miR-103 attenuated the growth rate of gastric cancer cells significantly in vitro and in vivo. Moreover, the invasion and metastasis ability of gastric cancer cells was also inhibited in vitro and in vivo. However, the apoptosis ability was promoted after transfection of miR-103 inhibitor. These results suggest that miR-103 plays a crucial role in promoting GC progression.

Overexpression of miR-103 has been found in several type human cancers and previous studies reported that miR-103 could enhance the proliferation, migration, and invasion of cancer cells by targeting anti-oncogenes *DICER* and *PTEN* [4], *TIMP-3* [5], *PDCD10* [6], *KLF4* [11] respectively. Despite the importance of miR-103 in development of cancer, the exact function of miR-103 in the progression of GC, especially with regard to migration and invasion, remained largely unknown, and its underlying molecular mechanisms have not been sufficiently elucidated.

Epithelial-to-mesenchymal transition (EMT) is an important process in cancer cell invasion and metastasis. EMT induction is driven by complicated molecular mechanisms. The role of miR-103 in EMT is less investigated. In our study, downregulation of miR-103 in SGC7901 and BGC823 cells showed a more epithelial-shaped phenotype, increased expression level of E-cadherin and decreased vimentin expression. EMT-associated genes tested, including *Twist*, *Snail*, *Slug* and *Zeb2*, were also downregulated by miR-103 knockdown. These results provide new proof that miR-103 promotes EMT of GC cells. Therefore, miR-103 could be an important oncogene that promotes proliferation and metastasis in GC.

To demonstrate the underlying mechanism of miR-103’s action in gastric cancer, we confirmed Krüppel-like Factor-4 (*KLF4*) as a direct target of miR-103. *KLF4* is a member of the KLF family of zinc-finger transcription factor. *KLF4* is overexpressed in the epithelium of the gastrointestinal tract and is associated with cell proliferation, growth arrest and late-stage cell differentiation [12,13]. Previous study reports that miR-103 promotes endothelial maladaptation and atherosclerosis by mediating suppression of *KLF4* [11]. *KLF4* has been confirmed as both anti-oncogene and tumor promoter in different types of cancer [14,15,16,17]. In GC, the function of *KLF4* has been characterized as tumor suppressor and serves as a prognostic predictor for the survival of patients [18]. Overexpression of *KLF4* significantly inhibited gastric cancer cell proliferation, invasion and metastatic properties [19]. This study identified *KLF4* as a direct target of miR-103 in GC through dual-luciferase reporter and confirmed by western blot analysis. Furthermore, an inverse association between miR-103 and *KLF4* expression in GC was observed. These results demonstrated that upregulation of miR-103 might promote GC cell proliferation, migration and invasion through the KLF4-mediated signal pathway.

Taken together, this study revealed that miR-103 was overexpressed in GC and associated with tumor size and lymph node metastasis. These results showed that miR-103 could facilitate cell proliferation, migration and invasion and reduce apoptosis. To investigate a direct targeting mechanism, *KLF4* was identified as a direct target of miR-103. miR-103 functions as an oncogene by inhibiting its target gene *KLF4* in gastric cancer. In conclusion, our results demonstrated that upregulation of miR-103 might contribute to gastric cancer progression by suppressing *KLF4* expression and subsequently promoting the proliferation, migration, and invasion, and inhibiting apoptosis of gastric cancer cells.

## 4. Materials and Methods

### 4.1. Tissue Specimens

Gastric cancer tissues (T) was obtained from 92 patients who underwent primary surgery of GC between March 2012 and February 2014 at Weifang People’s Hospital and Affiliated Hospital of Weifang Medical University. Adjacent non-tumor tissues (ANT) located at least 5 cm from tumors were selected randomly from 20 of these patients and used as controls. These 20 GC tissues and matched ANT were used for western blot analysis. Tissue samples were snap-frozen in liquid nitrogen after resection until use. The basic clinical characteristics of these patients were documented in Table 1. Patients’ consent and approval from the Ethics Committee of Weifang Medical University were obtained (identification code: 2012-012, date of approval: January 2012).

### 4.2. Cell Culture and Transfection

Human gastric cancer cells lines BGC823, SGC7901 and MKN45 were used in this study. MKN45 was obtained from the American Type Culture Collection (Manassas, VA, USA). BGC823 and SGC7901 were purchased from Shanghai Cancer Institute (Shanghai, China). All cell lines were cultured in RPMI 1640 medium (Hyclone, Logan, UT, USA) supplemented with 10% fetal bovine serum (FBS) (Gibco-BRL, Invitrogen, Paisley, UK). Cells were cultured at 37 °C in a humidified atmosphere containing 5% CO_2_. The miR-103 inhibitor and the corresponding negative control (miR-NC) were purchased from GenePharma (Shanghai, China). *KLF4* siRNA (si-*KLF4*) and the negative control (si-NC) were obtained from ambion (Austin, TX, USA). Transfection was performed using Lipofectamine 2000 Reagent (Invitrogen, Carlsbad, CA, USA) following the manufacturer’s instructions.

### 4.3. RNA Extraction and Real-Time PCR (qRT-PCR)

Total RNA was extracted from tissue samples and cell lines using Trizol (Invitrogen) according to the manufacturer’s protocol. SYBR^®^ Premix Ex Taq™ II kit (TaKaRa, Dalian, China) was used to quantify the expression levels of mature miR-103, EMT-associated genes and *KLF4* according to the protocol provided. Relative expression of miR-103 EMT-associated genes and *KLF4* was measured using the comparative cycle threshold (*C*_t_; 2^−ΔΔ*C*^^t^) method (primer sequences are given in Table 2). All reactions were run in duplicate.

### 4.4. Cell Proliferation Assay

Cell proliferation was determined using Cell Counting Kit-8 (CCK-8) solution (Dojindo, Gaithersburg, MD, USA) and the cell-light 5-ethynyl-20-deoxyuridine (EdU) Apollo Imaging Kit (Ribobio, Guangzhou, China) in accordance with the manufacturer’s protocol. For CCK-8 assay, cells were seeded at a concentration of 4 × 10^3^ cells/well into three replicate wells on a 96-well plate and treated with 10 μL/well of CCK-8 solution during the last 4 h of culture daily for four consecutive days. The absorbance at 450 nm was measured with a microplate reader. The EdU assay was performed according to manufacturer’s instructions. After EdU incubation, cells were treated with 1× Apollo solution and then stained with Hoechst 33342 (Ribobio, Guangzhou, China). The EdU positive cells was visualized under a fluorescence microscope (Olympus, Tokyo, Japan) and the positive percentage was defined as the proliferation rate. All reactions were performed in duplicate.

### 4.5. Flow Cytometry Apoptosis Assay

Cell apoptosis was measured by FITC Annexin V Apoptosis Detection Kit II (BD Bioscience, Woburn, MA, USA) following the manufacturer’s protocol. Cells were collected at 48 h after transfection and stained with propidium iodide and Annexin V-FITC solution in the dark for 15 min. Stained cells were detected by FACS Caliber flow cytometer (BD Bioscience) for early and late apoptosis analysis. All data were obtained from three independent experiments.

### 4.6. Cell Migration and Invasion Assay

To assess the migratory and invasive potential of cells in vitro, the migration and invasion assays were performed using transwell chambers with 8 μm pore (Corning star, Lowell, MA, USA). Briefly, 1 × 10^5^ transfected cells were suspended in 200 μL serum-free medium and added to the upper chamber of transwell chambers. After incubation for 24 h in a humidified atmosphere containing 5% CO_2_ at 37 °C, these migrated cells that had stuck to the lower surface of the membrane were fixed in 4% paraformaldehyde and stained with 0.1% crystal violet for 15 min. The number of migrated cells was counted from five randomly selected fields at 200× magnification using a microscope. For invasion assay, the transwell chambers were coated with matrigel (BD Bioscience) and other procedure is the same as migration assay. Each experiment was performed in triplicate.

### 4.7. Vector Construction and Luciferase Reporter Assay

The 3′-UTR sequence of *KLF4* or the mutant (Mut) was cloned into the downstream of the firefly luciferase gene in the pmirGLO plasmid (Promega, Madison, WI, USA). miR-103 inhibitor and miR-NC and the report plasmid pmirGLO-KLF4 Wt/Mut were co-transfected into cells (SGC7901 and BGC823) using Lipofectamine 2000 (Invitrogen, Carlsbad, CA, USA). After 48 h of transfection, luciferase activities were detected by Dual-Luciferase Reporter System (Promega, Madison, WI, USA) and Renilla-luciferase activities were measured for normalization. All experiments were performed three times.

### 4.8. Western Blot Analysis

At 48 h after transfection with miR-103 inhibitor or miR-NC, western blot analysis was done. Total protein was isolated from cells or tissues using RIPA buffer (Beyotime, Haimen, China) and was separated by SDS gels and then transferred to the nitrocellulose membranes. Immunoblotting was performed using the appropriate primary antibodies and the band was visualized using enhanced chemiluminescence reagents ECL (Pierce, Rockford, IL, USA). The following commercial antibodies were used in this study: KLF4 (1:1000, Proteintech, Wuhan, China), E-cadherin (1:1000, Santa Cruz, CA, USA), vimentin (1:1000, Santa Cruz) β-actin (1:1000; Cell Signaling, Danvers, MA, USA). Data were obtained from three independent experiments.

### 4.9. Xenograft Model Experiment

All animal experiments were performed according to institutional and international animal regulations. Animal protocol was approved by the Institutional Animal Care and Use Committee of Weifang Medical University (identification code: 2013-015, date of approval: February 2013). Male severe combined immunodeficiency (SCID) mice (four weeks old) were purchased from Beijing HFK Bioscience CO., Ltd. (Beijing, China). SGC7901 cells were transfected with lentivirus miArrest™ miR-103 inhibitor (Genecopeia, Rockville, MD, USA). SGC7901 cells were subcutaneously injected (1 × 10^6^ cells per mouse) and through tail vein into the mice (2 × 10^6^ cells per mouse), respectively. The growth of tumors was measured every seven days. All mice were euthenized after five weeks (subcutaneous xenograft group) or eight weeks (lung metastases xenograft group), and the tumor nodules and lungs of the mice were removed. Tumor sizes were measured using a caliper, and tumor volume was calculated according to the following equation: tumor volume (mm^3^) = length (mm) × width (mm)^2^/2. Histological analyses were used to detect distant metastasis in lungs in H&E sections. A portion of tumor tissues was used for analysis of RT-qPCR.

### 4.10. Statistical Analysis

All the data were presented as means ± standard deviation (SD). Statistical analyses were performed using Prism 5.01 ware (GraphPad Software, San Diego, CA, USA). Differences were determined with the analysis of variance (ANOVA), Student’s *t*-test, or a χ-square test as appropriate. The correlation between miR-103 level and tumor size or *KLF4* level was analyzed by Spearman analysis. Survival curves were plotted by the Kaplan-Meier method and analyzed by the log-rank test. *p* < 0.05 was regarded as statistically significant in all cases.

## Figures and Tables

**Table 1:** Association between the clinicopathologic parameters and miR-103 expression in gastric cancer (GC).

Characteristics	Number	Median	*p* Value
Age (years)	92		
≥60	52	0.6181	0.1022
<60	40	0.3569	
Gender			
Male	54	0.5837	0.2351
Female	38	0.3920	
Tumor size (cm)			
<5	44	0.3377	0.0429 *
≥5	48	0.6575	
Lauren’s classification			
Intestinal	52	0.3247	0.0088 *
Diffuse	40	0.7383	
Differentiation status			
Well/moderate	45	0.6385	0.0979
Poor	47	0.3762	
TNM stage			
I + II	32	0.3635	0.1948
III + IV	60	0.5797	
Lymph node metastasis			
Negative	36	0.3098	0.048 *
Positive	56	0.6297	

* *p* < 0.05 was considered significant.

**Table 2:** Nucleotide sequences of the primers used for RT-PCR in this study.

Human Gene	Primer Sequence 5′ to 3′	
	Forward	Reverse
*E-cadherin*	CGAGAGCTACACGTTCACGG	GGGTGTCGAGGGAAAAATAGG
*Vimentin*	GACGCCATCAACACCGAGT	CTTTGTCGTTGGTTAGCTGGT
*Twist*	GTCCGCAGTCTTACGAGGAG	GCTTGAGGGTCTGAATCTTGCT
*Snail*	TCGGAAGCCTAACTACAGCGA	AGATGAGCATTGGCAGCGAG
*Slug*	CAACGCCTCCAAAAAGCCAA	ACTCACTCGCCCCAAAGATG
*KLF4*	TCGGACCACCTCGCCTTACA	CTGGGCTCCTTCCCTCATCG
